# Observing the nature of relationships in male bottlenose dolphins

**DOI:** 10.1007/s10071-022-01672-y

**Published:** 2022-08-31

**Authors:** Wendi Fellner, Heidi E. Harley, Barbara A. Losch

**Affiliations:** 1 The Seas, Epcot®, Walt Disney World® Resort, Lake Buena Vista, FL 32830 USA; 2 Division of Social Sciences, New College of Florida, Sarasota, FL 34243 USA

**Keywords:** Affiliation, Synchrony, Association index, Male pair bonds, Bottlenose dolphin, Tursiops truncatus

## Abstract

As long-term studies reveal, bottlenose dolphin communities comprise a complex network of individual relationships. Individuals form strong bonds (e.g., mother-calf or male partnerships), transient relationships, and also compete against each other for resources. Evidence of bonded partnerships is typically revealed by the years-long study of associations with repeated sightings. However, quickly determining which individuals have close affiliations would benefit both field researchers working to describe individual behavior as they engage in cognitive activities such as cooperative foraging as well as caregivers in zoos who must decide which individuals should be housed together. Observations in aquariums are well-suited for collecting long-term, detailed information on how pairs interact because subjects can always be found and their behavior both above and below the water can be seen well. These are conditions that are rare for most (but not all) ocean-based studies. We used multiple measures to detect affiliated behavior across several dimensions of pairwise affiliation. Specifically, we used association indices to measure the frequency of affiliative behavior, the symmetry of the partnership, the tenor of interactions, and the stability of which partners were strongly affiliated from year to year. Synchronous behavior and reciprocity in proximity-seeking are two examples of potential markers of an affiliative relationship where individual choices–to join, to move together, and to leave–are visible to observers. We found that the combined measures were effective at identifying one pair that maintained a strong, stable relationship across years, one individual that formed a moderately strong trio relationship with both members of the most-affiliated pair, and one individual who was more variable in his relationships. These social markers provide a means of rapidly identifying bonded males in both aquarium and ocean settings, particularly when long-term knowledge of individual histories is not available.

## Introduction

Bottlenose dolphins (*Tursiops* sp.) are highly social animals who live in a fission–fusion society in which some individuals associate more frequently than others within a stable wider social network (Connor and Wells 2000; Wells 2014). For mother-calf pairs, the importance of calves’ multi-year associations with their mothers is well-established (Wells 2009). Adult males also form partnerships that last for at least decades if not lifetimes (Connor and Wells 2000; Owen et al. 2002; Wells 2014). Having a bonded partner appears to be the default condition in Sarasota Bay, FL (*T. truncatus*) as most unpaired males are either young and therefore have not yet formed a partnership or have lost a partner and not yet found a new one (Owen et al. 2002). The presence of a stable partner or partners can impact several cognitive processes that are relevant for survival: cooperation while foraging (Wells 2019; and for the related spinner dolphin species, *Stenella longirostris*, Benoit-Bird and Au 2009), social learning (Fellner et al. 2006; Sargeant and Mann 2009), reproductive success (Gerber et al. 2022), and vocal copying which likely is important to maintaining relationships that are advantageous (Smolker and Pepper 1999; Watwood et al. 2004; King and Janik 2013; King et al. 2013). Knowing which males are bonded and which are not is an important factor to consider when investigating these vital cognitive phenomena because how close your partnership is may influence whether you partake in these activities, how skilled you are at performing them together, and which roles are taken.

In an ocean setting, it is difficult to determine which individuals have long, stable relationships without years of ongoing observations. A common way to determine the strength of relationships between individuals is to calculate an association index, such as the half-weight index of association (HWI) or Simple Ratio Index (SRI) in which 10–20 sightings per individual are used to determine how often two individuals are seen together in a group compared to seen without each other (e.g., Wells et al. 1987; Quintana-Rizzo and Wells 2001). It can take several years to accumulate a sufficient number of sightings per individual to produce an accurate index that will distinguish close associates from casual associates from animals that do not associate with each other at all (e.g., Frere et al. 2010).

In aquarium facilities, managers and care staff must make decisions about which individuals can be successfully housed together in such a way as to optimize their welfare. Finding individuals that are compatible is relatively straightforward by evaluating the lack of aggression but may not result in optimal groupings in which the animals form a true bonded partnership. Time spent near each other may reflect a level of tolerance or independent shared interest in the location without indicating which partners would choose to be together.

To address these needs in both environments, we sought to develop a method of detecting which animals have formed bonded partnerships and which are more casually associated with each other. We based our assessments on the detailed, long-term observations that can most easily be done in an aquarium setting in which the animals are always accessible and the visibility is always clear. Once relationship status was determined using these fine-resolution methods, we looked for defining characteristics of those relationships that may be accessible more quickly and with reduced viewing opportunities.

Silk et al. (2013) put forth the idea that a richer understanding of relationships can be gained using multiple measures that apply to diverse dimensions of dyadic interactions. Many studies depend upon frequency as their primary measure (i.e., how much time do individuals spend near each other), but frequency alone may not be sufficient to understand some dynamics of a relationship such as which is more responsible for maintaining the contact? How often is the contact affiliative and how often is it agonistic? Is the relationship long-standing or contextual? Silk et al. (2013) proposed jointly deploying several association indices to describe different aspects of dyadic relationships as a way of obtaining a more robust understanding of their nature. We used this framework to assess relationships in male dolphins along four of their seven proposed dimensions, which we will describe more fully below. The four dimensions that could be assessed with our data set included frequency, symmetry, tenor, and stability. Frequency refers to how often an affiliative act or state occurs. Symmetry is a measure of whether the affiliative behaviors are one-sided or reciprocal. Tenor is the balance of friendly or cooperative acts as compared to hostile or aggressive behavior. Stability assesses whether most favored partners are the same from year to year or vary.

One behavior often associated with affiliation in dolphins is synchronous movement. Synchronous movement is by definition a social behavior and has been described in a variety of contexts. For mother-infant pairs, synchrony may serve to enhance the calf’s energy conservation through slipstreaming, provide protection through close proximity, optimize efficient positioning for feeding, and potentially serve as a platform for social learning throughout early development (Fellner et al. 2006). Males in alliances that herd females together (*Tursiops aduncus*) are more likely to breathe synchronously (often used as a proxy for synchronous movement when water is turbid), but males from second-order alliances (and therefore weaker associations) have tighter synchronization than do first-order alliance partners, suggesting a complex relationship between the precision of synchrony and relationship strength (Connor et al. 2006; McCue et al., 2020). In a mixed-species setting, smaller Atlantic spotted dolphins (*Stenella frontalis*) create a unified group by adopting synchronous postures to defend themselves against larger common bottlenose dolphins (Cusick and Herzing 2014; Myers, Herzing et al. 2017). As synchrony is common among males in multiple contexts, we chose synchrony as an appropriate lens through which to explore the strength of long-term affiliative relationships.

Here we present the results of long-term, detailed observations of a population of male dolphins with known life histories living in an environment of perfect visibility. Our goal is to develop effective methods that identify which males in a group are closely bonded. We chose multiple measures as described below that each assesses unique aspects of affiliation to produce a rich representation of each dolphin’s relationship with the other dolphin. We sought to identify the strength of relationships among each pair, assess how stable those bonds remain over a period of 16 years, and suggest which measures may be used to detect the bonded relationship in a short period of time.

## Methods

### Subjects and location

**Fig. 1:**
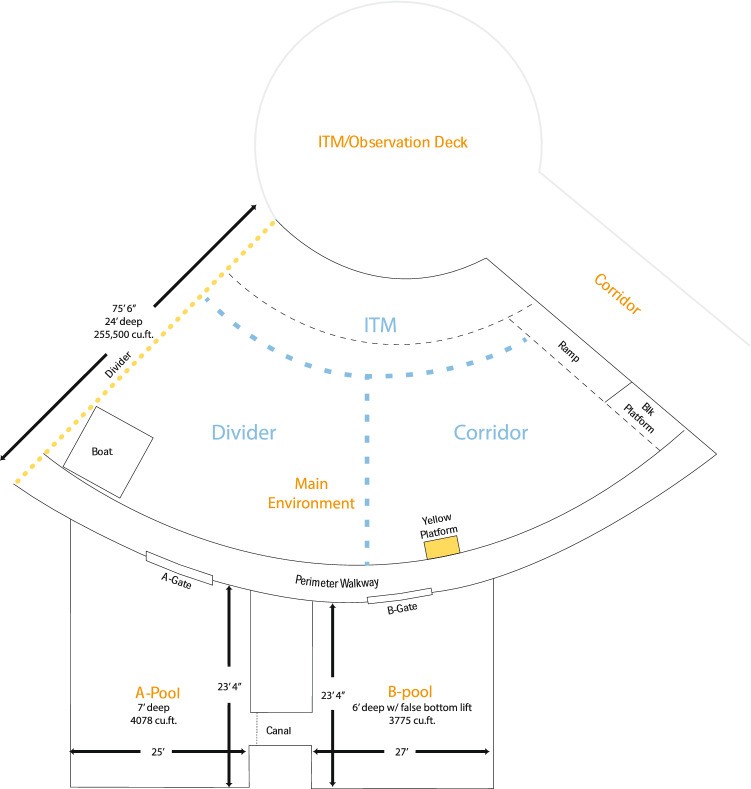
A diagram of the dolphins’ primary habitat. The large Main Environment was an open area divided into three named locations for this study using visual landmarks. A dolphin was recorded as being in a location if more than half his body was located within it at the moment of instantaneous sampling

The subjects were all four of the bottlenose dolphins (*Tursiops truncatus*) that resided in one-quarter of a 5.8 million-gallon, mixed-species exhibit and two adjoining pools at The Seas, Epcot®, Walt Disney World^®^ Resort, Lake Buena Vista, FL, USA (see Table 1 for names, ages, and history of each dolphin). The Seas is open year round and does not close for an off-season. The primary area available to the dolphins comprised three pools that were connected by underwater swim-through gates (Fig. 1) The gates were open most of the time so the dolphins could choose their location. On five occasions (2014, 2016, 2017, 2018, and 2021), maintenance activities in their primary habitat required temporary relocation to other pools for periods of one to four months, and this study was conducted in all of the sites where the dolphins live. Their behavior in relation to each other remained consistent with what we had observed in the main environment. The dolphins were typically managed as a single group of four but were sometimes divided into pairs for periods of time ranging from a few hours to several months. This led to differing numbers of observations for each pairing as not all pairs were always available to each other. The dolphins regularly participated in five varied sessions per day that included husbandry, informal interactions with trainers, cognitive research sessions that also served as guest presentations through underwater windows, and one guest interaction per day (see Harley et al. 2010 for more information about the kinds of activities that made up the dolphins’ day). Each dolphin consumed a diet of herring, capelin, and squid that was customized for each individual by the nutritionists, veterinarians, and trainers on the Animal Health and Animal Care teams, and who were responsible for all care and management decisions. Disney’s Animal Care and Welfare committee reviewed and approved the project (IR1005 and IR1809). The dolphins were cared for in accordance with the U.S. Animal Welfare Act at all times (USDA display permit 58-C-0076) as well as the accreditation guidelines of the Association of Zoos and Aquariums.

**Table 1:** Names and individual characteristics of the common bottlenose dolphin (*Tursiops truncatus*) subjects

Dolphin	Sex	Birth year (location type)	Rearing History	Arrival at The Seas	Disposition
Calvin	M	1994 (sea pen)	Parent/adopt/hand	2003	Remains at The Seas
Khyber	M	1992 (sea pen)	Parent-raised	2005	Died 2018
Malabar	M	2000 (sea pen)	Parent-raised	2005	Remains at The Seas
Ranier	M	1981 est (Gulf of Mexico)	Parent-raised	2001	Remains at The Seas

Observations began in September 2005 when Calvin was 11, Khyber was 13, Malabar was 5, and Ranier was estimated to be 24 years old

### Synchrony

Observations began with Calvin and Ranier in September 2005, three months prior to the arrival of Khyber and Malabar, and continued for four years through September 2009. Data collection resumed in August 2013 through December 2021. In two years (2007 and 2013), the group was divided into two consistent pairs, so only observations of those pairs are reported for those years. We observed each dolphin for 7.5 min (31 observation points), 1–2 times per day, 5–7 days per week using a focal animal, instantaneous sampling technique (Altmann 1974) and selected each session’s focal animal by following a randomized schedule. Two of us (WF and BL) completed independent practice observations until achieving 90% concordance or better. BL then served most frequently as the observer and also trained all additional observers using similar procedures. The observer recorded the focal animal’s location every 15 s throughout the observation, and also described his synchrony status, position, and proximity in relation to each available partner, whether any of the other dolphins were in the same location as the focal animal, and whether each breath was solo or synchronized with a partner (see Table 2 for definitions). A 15-s interval was chosen because it was long enough to enable recording data in real time but short enough to reliably capture brief behavioral states with few omissions.

**Table 2:** The definitions of synchrony, positions, and proximity follow those of Fellner et al. (2013) and illustrations therein

**Synchrony Status**	
Synchronous movement	Moving together while oriented in the same direction, regardless of proximity or position
**Position**	
Echelon	Side-by-side with one dolphin’s rostrum no farther behind than the other’s trailing edge of the dorsal fin
Genital leading/genital following	One slightly trailing the other with one’s rostrum falling into a region from the other’s dorsal fin to the insertion of the flukes (analogous to Infant position in Fellner et al. (2013))
Leading/following	One trailing the other with one’s rostrum behind the insertion of the other’s flukes
**Proximity**	
Touching	Direct contact
Near	Within one adult dolphin width (~ 18″)
Intermediate	Within one adult dolphin length (~ 9’)
Far	Farther than one adult dolphin length
**Location**	
Focal’s location	Within a named location (Fig. 1, e.g., “A-Pool”)
Same location	Within the same named location as the focal dolphin
**Synchronous breath**	
Synchronous breath	A breath taken within 1 s of another dolphin, regardless of synchronous movement status

We assessed the strength of each pair’s affiliative relationship under the Silk et al. (2013) framework by considering the *Frequency* of synchrony and association, the *Symmetry* in approaching and leaving each other, the *Tenor* expressed by positive and negative interactions, and the *Stability* in most frequent synchrony partner from year to year. We also assessed monthly and seasonal (as demarcated by the solstices and equinoxes) variability in synchrony averaged across years, and we calculated z-scores to determine if any month or season was significantly different from the mean. Throughout, we considered each dolphin’s behavior in relation to each other dolphin in the population which yielded dyadic information. That is, to describe the Calvin-Ranier pair, we combined observations for Calvin as the focal animal in relation to his behavior with Ranier and observations for Ranier as the focal animal in relation to his behavior with Calvin. Throughout this paper, if the formula for an index calls for “Partner A” and “Partner B”, Partner A is always reported as the first named individual in a described pair and Partner B as the second.

#### Frequency

We calculated the proportion of synchrony as the number of points the pair was seen in synchrony divided by the total number of observation points in which both animals were seen, times 100, calculated annually and overall. A half-weight index of association (HWI; Hoppitt and Farine 2017) for each pair was also calculated annually and overall by considering the number of times members of a pair were observed in the same location (e.g., A-Pool) divided by the total number of observations. The frequency of synchrony measure ranges from 0 to 100% and HWI returns values between 0 and 1 with higher numbers indicating more frequent synchrony or more frequent association between partners. We included only times when both members of the pair were available to each other, and for the half-weight index, when at least two locations were available and so the individuals had the option to choose to be together or not.

#### Symmetry

To examine whether one member of a pair was more active in maintaining an association, we used Hinde’s Index of Association (Hinde and Atkinson 1970) which considers the ratio of each partner’s actions to come together or to leave the other. We calculated each partner’s “joins” and “splits” by comparing changes from one observation point to the following point 15 s later in the focal animal’s location and which other dolphins were in the same location. For example, if at time 0:00 focal Calvin was in A-pool and Malabar was not in the same area and then at time 0:15 Calvin was still in A-pool and now Malabar is also in A-pool, this would be tabulated as “Malabar joins Calvin”. Then at 0:30, focal Calvin is now in B-pool and Malabar is not in the same area, this would be tabulated as “Calvin splits from Malabar”. Hinde’s Index is calculated by taking the ratio of Partner A joining divided by all joins by Partner A + B and then subtracting the ratio of Partner A splitting divided by all splits by Partner A + B. The index returns a score between + 1 and − 1 with values closer to + 1 indicating Partner A is maintaining the association and values closer to − 1 indicating Partner B is maintaining the association. A score at the extremes of the scale is obtained when one partner consistently splits and the other partner consistently joins, but a middle value of 0 can be obtained in two scenarios: one partner both joins and splits exclusively, or both partners evenly share in joins and splits. Therefore, in the case of a near-zero result, we calculated Brown’s Index (Brown 2001) which indicates whether one partner is more active in changes in space-sharing or both partners are equally active. Brown’s index is a ratio of Partner A’s joins + splits divided by all joins and splits by both partners and then multiplying by 100. The index returns a score between 0 and 100 with scores closer to 100 indicating Partner A is generating all the join/split activity and may simply be more active in moving around than the other with neither actively seeking out or retreating from the other. Scores closer to 0 indicate Partner B is more active, and scores near 50 indicate equal activity. We calculated symmetry scores for each pair annually and overall.

#### Tenor

The overall tenor of a relationship can be described by comparing the frequency of friendly or cooperative behavior to agonistic or hostile behavior. Although we did not record overt agonistic behavior directly, we hypothesized that specific synchrony positions may be expressed differentially between pairs with different relationships. Echelon position is side-by-side and may imply an equality between partners. In contrast, when one dolphin is leading or following the other, we thought this to be less likely to be affiliative as sometimes this behavior leads to chasing which may be experienced more negatively. We calculated a Relationship Quality Index (Perry et al. 2004) in which the number of echelon observations was divided by the total number of echelon, following, and leading observations, yielding a score between 0 and 1. A score of 0 indicates pairs that have only negative (i.e., following) interactions, and a score of 1 only friendly (i.e., echelon) interactions. For comparison, we also calculated the Relationship Quality Index using non-synchronous proximity in which the number of touching and near observations was divided by all proximities. We calculated these indices for each pair annually and overall.

#### Stability

To assess the stability of each pair’s relationship, we determined each dolphin’s most frequent synchrony partner for each year and then tabulated the maximum number of consecutive years in which the top partner and the “not top partner” was the same as the previous year (Silk et al. 2013). Ties were assigned “top” status for both partners, zero synchrony was assigned “not top” for all partners, and years without data were not included. Higher numbers indicate a more stable relationship.

To explore the potential influence of circannual cycles related to seasonality or breeding motivations, we calculated z-scores to assess variability in monthly and seasonal synchrony rates collapsed across years. Seasons were as designated for the northern hemisphere and divided in alignment with the spring and fall equinoxes and summer and winter solstices.

Many field sites have turbid water and so dolphins are only visible when surfacing for a breath. For this reason, it is common to use synchronous breaths as a proxy for synchronous movement. We examined the relationship between breathing and motor synchrony by calculating the correlation coefficient between the proportion of time swimming synchronously and the number of breaths taken synchronously with a partner, whether moving synchronously or not, during each 7.5-min observation.

Because our sample size included only four individuals and the measures were not independent of each other, we report only proportions, raw scores, and the correlation coefficient between variables. For correlation coefficients, |*r*|< 0.2 is interpreted to be unrelated, 0.2 >|*r*|< 0.4 is weakly or low-moderately related, 0.4 >|*r*|< 0.6 is moderately related, 0.6 >|*r*|< 0.8 is strongly related, and |r|> 0.8 is very strongly related, with the sign of the *r* value indicating whether the relationship between variables is positive or negative.

## Results

### Overall affiliation: 2005—2021

All pairs demonstrated synchrony behavior, and overall rates differed between pairs (range: 2% for Khyber/Ranier to 19% for Calvin/Ranier; Table 3). The half-weight index based on location-sharing ranged from 0.29 (low-moderate for Khyber/Malabar) to 0.52 (moderate for Malabar/Ranier). When considering symmetry, Hinde’s index revealed that in all pairs, neither partner was more responsible than the other for being in a shared location as all scores were near zero. Brown’s index indicated that Khyber was slightly more active than Ranier in entering and leaving shared spaces (Brown’s = 65), that Calvin was slightly more active than Ranier (Brown’s = 62), and that Calvin was slightly more active than Malabar (Brown’s = 60), but that both partners were equally active in the other three pairings (range: 48–56). The Relationship Quality Index based on echelon and following synchrony returned all positive tenor relationship values from 0.68 for Malabar/Ranier to 0.96 for Calvin/Ranier. In contrast, the same index based on proximity while not synchronous ranged from 0.05 (Khyber/Malabar) to 0.11 (Calvin/Malabar and Malabar/Ranier), suggesting a negative tenor for all.

**Table 3:** Two indices each applied to the dimensions of frequency, symmetry, and tenor

	Frequency		Symmetry		Tenor	
	Sync	HWI	Hinde’s	Brown’s	RQI: Sync	RQI: Proximity
Calvin/Khyber	7%	0.33	0.00	56	0.83	0.07
Calvin/Malabar	8%	0.47	− 0.01	60	0.72	**0.11**
Calvin/Ranier	**19%**	0.51	0.01	62	**0.96**	0.07
Khyber/Malabar	7%	0.29	− 0.02	56	0.88	0.05
Khyber/Ranier	2%	0.37	0.02	65	0.75	0.06
Malabar/Ranier	6%	**0.52**	0.02	**﻿48**	0.68	**0.11**
Possible Index Range	0–100%	0–1	− 1 to + 1	0–100	0–1	0–1

Bold numbers indicate the result that reflects the strongest affiliation for that measure

For frequency and tenor measures, higher numbers suggest more affiliation, and for symmetry, middle numbers suggest more equality. The frequency of synchrony is the proportion of time moving synchronously over total observations. Half-weight index (HWI) is based on the proportion of times pairs were in the same location over all observations. Hinde’s is based on which individual joins and leaves the other with extreme values indicating one partner is more responsible for maintaining togetherness. Values near 0 must be disambiguated with Brown’s index to understand whether one partner is most active in achieving the median Hinde’s score. Values closer to 0 or 100 indicate only one partner joins and leaves, and values closer to 50 indicate both partners join and leave equally. Relationship quality index (RQI) is based on the proportion of echelon synchrony to following + echelon synchrony or based on the proportion of touching + near proximity to all proximities while not synchronous. The pair with the highest affiliation on each measure is bolded

When considering the entire time span, Calvin/Ranier had the most synchrony as well as the most positive tenor to their synchronous interactions. Malabar/Ranier had the highest half-weight index but was followed closely by Calvin/Ranier, the most equality in choosing to share space with each other and, tied with Calvin/Malabar, the most positive tenor in relation to proximity.

### Affiliation by year

**Fig. 2:**
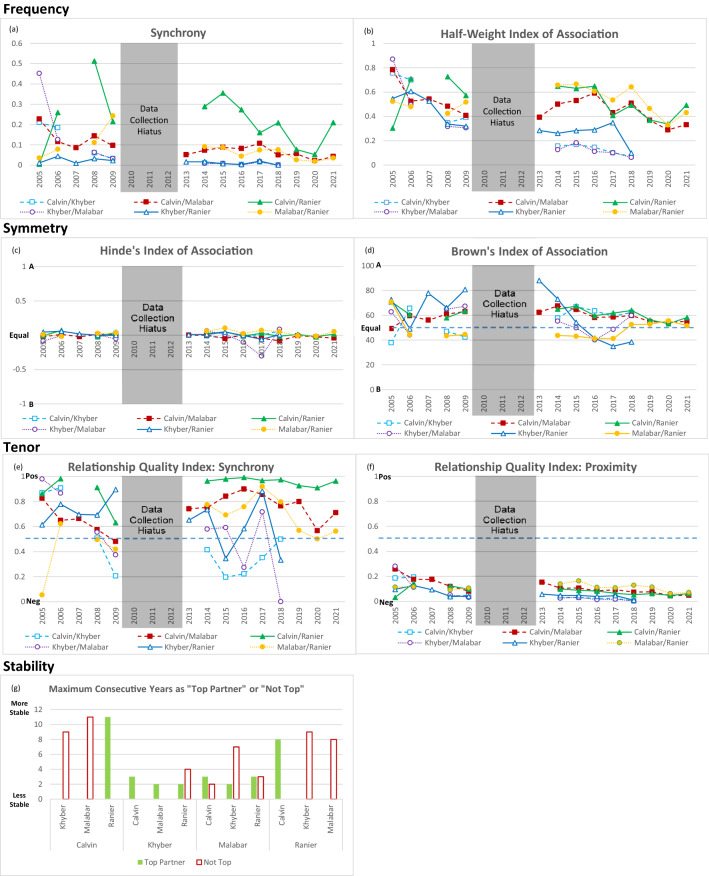
Annual values of each affiliation index. Frequency of affiliation is shown as (**a**) percentage of time engaged in synchronous behavior and (**b**) as a Half-Weight Index based on observations of partners being together in the same location. Symmetry is shown first by (**c**) Hinde’s Index for determining responsibility for sharing a location followed by (**d**) Brown’s Index which shows how active each partner is in contributing to the results of the Hinde’s Index. Tenor is shown by (**e**) the ratio of echelon synchrony over echelon + following synchrony and (**f**) the ratio of touching + near proximity over all proximities. Stability is shown by (g) the maximum number of consecutive years in which top and not top partners remain the same and (**a**–**f**) variability in the annual trends in the other indices. When determining stability scores in relation to “top” and “not top” status for synchrony partners, ties were assigned “top” for both partners, zero synchrony was assigned “not top” for all partners, and years without data were not included

The year Khyber and Malabar arrived at The Seas, 2005, was different from other years in multiple respects. For the four months that Calvin and Malabar were observed in 2005 and the six weeks after Khyber and Malabar’s November arrival, Khyber/Malabar were the most synchronous (45%), had the highest half-weight index score (0.87), and the highest relationship quality score based both on synchrony (0.98) and on proximity (0.28; Fig. 2). The pair that had been living together, Calvin and Ranier, displayed the lowest synchrony (0%), had the lowest half-weight index score (0.30), and the lowest relationship quality score based on proximity (0.03). However, in the following years, Calvin and Ranier consistently displayed the strongest affiliation in multiple measures. They had the highest rate of synchrony in all years except 2009 in which Malabar/Ranier slightly overtook them, had generally high half-weight index scores (range 0.34–0.73), and maintained a relationship quality index for synchrony above 0.90 for 10 of 11 years in which they had access to each other. When considering the stability of top synchrony partners, Calvin/Ranier were each other’s top partners for eight and eleven consecutive years, respectively, reflecting more stability than any other pair. Moderately stable top partners for three consecutive years include Calvin as Khyber’s partner, Calvin as Malabar’s partner, and Ranier as Malabar’s partner, but these top partner choices were not reciprocated. There was also stability in “not top partner” relationships. Malabar was not Calvin’s top partner in 11 consecutive years, Khyber was not Calvin’s or Ranier’s top partner for nine consecutive years, Malabar was not Ranier’s top partner for eight consecutive years, and Khyber was not Malabar’s top partner for seven consecutive years. Khyber’s top partners shifted from year to year, resulting in the lowest stability scores for both top and not top partners.

### Stability across months and seasons

Z-scores did not reveal a consistent pattern among pairs across months of the year or seasons. Only three months had synchrony of |*z*|> 2.0, and they were different months and different pairs (March =  + 2.46 for Khyber/Ranier, September =  + 2.41 for Calvin/Malabar, and November =  + 2.47 for Khyber/Malabar). Exclusive of those month-pairs, monthly variability ranged from *z* = − 1.65 to 1.65. Seasonal variability ranged from *z* = − 1.44 to 1.47. No months or seasons were paired consistently with each other in direction of being higher or lower than the mean.

### Motor and breathing synchrony

There was a moderate positive relationship between synchronous behavior and synchronous breaths for all pairs. R-values by pair ranged from 0.38 to 0.59.

## Discussion

### Affiliation identifiers per pair

The primary goal of this study was to identify reliable indicators of stable bonded relationships among male bottlenose dolphins. Using multiple measures provided a richer understanding of which pairs were most likely to be bonded beyond a simple measure of which were spending the most time together. Several of the measures returned results that were consistent with each other in ranking most affiliated to least affiliated pairs, providing a constellation of measures that could be used to infer bonded relationships with observations over briefer periods of time. Synchrony in particular was a powerful indicator of which animals will have the strongest relationships on a variety of measures.

Time spent in synchrony was in general agreement with the more common half-weight index in terms of ranking pairs in relation to each other but was also more sensitive in identifying one pair that was regularly synchronous two to three times more often than the next most synchronous pairs. To maintain synchrony requires joint coordination and therefore mutual consent or desire can be presumed in most cases, particularly in the echelon position. In addition, a snapshot taken in almost any year of the study (the introductory year excepted) would have returned the same results, which may be useful to field researchers in that it can take several years and thousands of surveys to accumulate the approximately 20 sightings of each individual needed to generate a reliable association index value (Hoppitt and Farine 2017; e.g., Frere et al. 2010). That behavioral synchrony and breathing synchrony are positively correlated imparts even more flexibility, as dolphins surfacing together are likely to be swimming together, although the two measures were not in perfect agreement. Assessing synchronous behavior should be preferred when the viewing conditions allow.

Assessing the symmetry of the relationship, that is to say its equality or reciprocity, is valuable in that it would identify instances in which one partner is dominating the other or other imbalances exist, as in hierarchies. This makes sense in developmental relationships (for which this index was developed) as a way to observe the gradual transfer of responsibility as offspring mature. It is also useful in this case of male relationships to understand whether the choice to join or leave a shared space reflects mutual attraction. The measure may be even more sensitive if it were based on which partner joins and leaves a synchronous bout of swimming (which was not possible using this instantaneous sampling method) because synchrony requires more coordination and intimacy than simply entering or leaving the same area. In instances in which one partner is attempting to flee another who then pursues and recaptures him or her, as in males pursing potentially unwilling females for consorting (Connor, Heithaus et al. 2001), symmetry scores would be near the extremes of + 1 or − 1. With minor exceptions, individuals within this group demonstrated equality for sharing locations which suggest that one dolphin was not actively avoiding another by leaving a space when the other approached, nor dominating another by pursuing whenever the other left.

The biggest discrepancy between measures occurred in the dimension of tenor. The relationship quality index based on proximity failed to differentiate between individual pairs and reflected a very low proportion of being in close physical proximity. It is possible that the proximity distances chosen were not biologically meaningful to these dolphins because they can maintain acoustic contact with each other at every distance within the facility (Quintana-Rizzo et al. 2006). Therefore a “far” distance in our coding scheme may not be the same thing as “distant” because the two can maintain direct communication no matter their location within the environment. In contrast, the tenor based on synchrony was variable from year to year but nonetheless retained the same overall pattern of consistently showing Calvin/Ranier as having the most positive tenor, Malabar having a mostly positive tenor with both Calvin and Ranier, and Khyber being most variable. However, it should be noted that the “following” position was assumed to be non-affiliative and not a direct measure of “chasing”. This measure could be further refined by assessing the frequency of affiliative and agonistic behaviors directly.

Annual stability scores take longer to determine because observations over multiple years are needed, but the results here align well with other measures that can be gathered more quickly. Our results indicate that those partners with the highest frequency of synchrony are also likely to demonstrate stability if observed over time. On shorter time scales, one could potentially extrapolate from monthly stability scores calculated over one year as our results suggest that highly synchronous pairs may remain stable over longer time periods. Calvin and Ranier show both longevity and reciprocity in their status as each other’s top partners. Malabar does not have a reciprocal top partner, but top partner status is evenly shared with Calvin and Ranier, forming a moderate trio relationship. Khyber’s top partner is nearly even among the three, indicating instability in his associations.

Although there was variability in synchrony, the lack of a consistent annual pattern (monthly or seasonal) suggests that strong affiliative bonds are advantageous year-round and not solely related to mating or other seasonal activities. However, different results may be found in mixed-sex settings or environments with more seasonal variability in lighting, water temperature, and prey availability. Environmental cues to the changing of seasons may portend greater or lesser needs for individuals to strengthen their close associations, associations that may directly affect their ability to protect themselves from predators or to enhance foraging success. Therefore, dolphins living in ocean environments may display more regular variability, which should be investigated.

One aspect that was not investigated here was the role of agonistic behavior in long-term, bonded relationships and within a community. Future work could consider adding a measure of agonism, such as receiving “rake marks” from another dolphin’s teeth, to the other association indices presented here to provide an even more robust assessment of multiple aspects of male relationships. Raking occurs during social encounters and is assumed to be the result of aggression (e.g., Clegg et al. 2015; Lee, Wallen et al. 2019) and related to competition (Hamilton et al 2019), but to our knowledge, no one has compared the acquisition of rakes to measures of affiliation. It could be that having a strong affiliative partner protects against receiving rakes, or that some number of rakes is inevitable in social activity, perhaps even suggesting a positive social life.

### Applications

Many of the measures considered here could be useful for quickly assessing which individuals are likely to have stable affiliative bonds when only brief observations are possible. For example, two dolphins that are surfacing or swimming synchronously, have equality in which partner leaves or joins the other, and swim frequently in echelon formation with low incidences of following are likely to have a bonded relationship that is stable. Synchronous behavior or breaths, momentary leaves and joins, and echelon and following behavior can be detected within a sighting, even in turbid water. Having a way to quickly detect this important relationship would enable the ability to ask relationship-based questions such as, “are bonded males more likely to fish-whack to each other than males that aren’t bonded?” or “how often do non-bonded males vocally copy each other?” Using these techniques may make it possible to ask these questions in populations for which long-term association data aren’t available.

As for managed care, it is important to consider that these stable relationships formed in an environment free of predators and with an ample food supply. That is, close associations formed despite the lack of direct environmental pressure for survival. For males, having a bonded partner is a natural and important part of their social organization, and assessing their relationship status should be an equally important part of their care. Using multiple measures to assess affiliative behaviors and identify bonded male partners provides rich knowledge of individual relationships that should be integral to managing populations.

## Data Availability

The dataset generated and/or analyzed during the current study are available from the corresponding author on reasonable request.
